# Classification of Alzheimer’s Disease by Combination of Convolutional and Recurrent Neural Networks Using FDG-PET Images

**DOI:** 10.3389/fninf.2018.00035

**Published:** 2018-06-19

**Authors:** Manhua Liu, Danni Cheng, Weiwu Yan, Alzheimer’s Disease Neuroimaging Initiative

**Affiliations:** ^1^Department of Instrument Science and Engineering, The School of Electronic Information and Electrical Engineering, Shanghai Jiao Tong University, Shanghai, China; ^2^Shanghai Engineering Research Center for Intelligent Diagnosis and Treatment Instrument, Shanghai Jiao Tong University, Shanghai, China; ^3^Department of Automation, The School of Electronic Information and Electrical Engineering, Shanghai Jiao Tong University, Shanghai, China

**Keywords:** Alzheimer’s disease diagnosis, FDG-PET, convolutional neural networks (CNN), recurrent neural network, deep learning, image classification

## Abstract

Alzheimer’s disease (AD) is an irreversible brain degenerative disorder affecting people aged older than 65 years. Currently, there is no effective cure for AD, but its progression can be delayed with some treatments. Accurate and early diagnosis of AD is vital for the patient care and development of future treatment. Fluorodeoxyglucose positrons emission tomography (FDG-PET) is a functional molecular imaging modality, which proves to be powerful to help understand the anatomical and neural changes of brain related to AD. Most existing methods extract the handcrafted features from images, and then design a classifier to distinguish AD from other groups. These methods highly depends on the preprocessing of brain images, including image rigid registration and segmentation. Motivated by the success of deep learning in image classification, this paper proposes a new classification framework based on combination of 2D convolutional neural networks (CNN) and recurrent neural networks (RNNs), which learns the intra-slice and inter-slice features for classification after decomposition of the 3D PET image into a sequence of 2D slices. The 2D CNNs are built to capture the features of image slices while the gated recurrent unit (GRU) of RNN is cascaded to learn and integrate the inter-slice features for image classification. No rigid registration and segmentation are required for PET images. Our method is evaluated on the baseline FDG-PET images acquired from 339 subjects including 93 AD patients, 146 mild cognitive impairments (MCI) and 100 normal controls (NC) from Alzheimer’s Disease Neuroimaging Initiative (ADNI) database. Experimental results show that the proposed method achieves an area under receiver operating characteristic curve (AUC) of 95.3% for AD vs. NC classification and 83.9% for MCI vs. NC classification, demonstrating the promising classification performance.

## Introduction

Alzheimer’s disease (AD) is a progressive and irreversible brain degenerative disorder which often affect the people older than 65 years. At present, there are around 90 million people who has been diagnosed with AD, and it is estimated that the number of AD patients will reach 300 million by 2050 (Zhan et al., 2015; Zhu et al., 2015). Mild cognitive impairment (MCI) is often considered as a clinical precursor of AD, which is a transitional state from normal control (NC) to AD dementia (Silveira and Marques, 2015). Currently, there is no effective cure for AD, thus the early detection at its prodromal stage and accurate diagnosis is vital for patient care and developing future treatment. In the past few decades, neuroimaging technologies have been widely used to discover AD-related biomarkers in human brain and help AD diagnosis. Positrons emission tomography (PET) is a functional molecular imaging modality through obtaining the virtue of focus glucose metabolic activity and distribution via imaging agent such as 18F-fluorodeoxy-glucose (18F-FDG) (Minati et al., 2009; Silveira and Marques, 2015). A positron-emitting radionuclide (tracer) with a biologically active molecule, such as (18)F-fluorodeoxy-glucose ((18)FDG), is introduced in the body. Concentrations of this tracer are imaged using a camera and indicate tissue metabolic activity by virtue of the regional glucose uptake (Silveira and Marques, 2015). It proves to be a powerful functional imaging tool to help physicians to diagnose AD. Therefore, Fluorodeoxiglucose positron emission tomography (FDG-PET) brain image becomes one of the powerful functional biomarkers for AD diagnosis in clinical and computer aided diagnosis.

In recent years, various pattern recognition methods have been investigated for analysis of FDG-PET brain images to identify the patterns related to AD and decode the disease states for computer-aided-diagnosis (CAD) (Cabral et al., 2013; Liu et al., 2014; Lu et al., 2015; Shen et al., 2017). A region based method was proposed to select the regions of interest, extract discriminate features and classify AD using PET brain images (Garali et al., 2016). In this method, brain images were mapped into 116 anatomical regions of interest (ROIs) and the first four moments and the entropy of the histograms of these regions are computed as the features. Receiver operating characteristics (ROC) curves are then used to rank the discriminative ability of ROIs to distinguish PET brain images. Finally, the features from top 21 regions are fed into both support vector machine (SVM) and random forest classifiers for AD classification. A ROI-based method was also proposed using FDG-PET images in (Gray et al., 2011), which segments the whole brain into 83 anatomical regions according to the MRI-space of each subject and extracts the mean signal intensity per cubic millimeter in each region from the FDG-PET images as the features. A SVM classifier was trained with these features for AD classification. In (Lu et al., 2015), 286 features were extracted from 116 cerebral anatomical volumes of interest (VOIs) based on the automated anatomical labeling (AAL) cortical parcellation map, and a semi-supervised method was proposed to integrate the labeled and unlabeled data by random manifold learning with affinity regularization for AD classification. In addition, a ROI-based method was proposed to combine the cross-sectional and longitudinal multi-region FDG-PET information for classification in (Gray et al., 2012). The regional image intensities were extracted as one kind of important features at each time point, as well as the changes of the image intensities over the follow-up period are combined for classification of AD.

Instead of extracting the features from ROIs, the voxel-wise intensity features were extracted to capture the rich feature after preprocessing PET images, including co-registration to their baseline PET scan, reorientation into a standard space, voxel intensity normalization and smoothing with a 8 mm FWHM Gaussian filter for AD classification. In (Silveira and Marques, 2015), a boosting classification method was proposed for classification of FDG-PET images based on a mixture of simple classifiers, which automatically performs feature selection concurrently with the classification to solve high dimensional problem. A favorite class ensemble of classifiers was proposed with each base classifier using a different feature subset (Cabral et al., 2013). Recently, deep learning methods can take the advantage of the data privilege to extract the latent features from measurements of ROIs with different image modalities for AD classification (Liu et al., 2015; Suk et al., 2015). Liu et al. (2015) extracted a set of latent features from 83 ROIs of MRI and PET scans and trained a multi-layered neural network consisting of several auto-encoders to combine multimodal features for image classification. Suk et al. (2015) used a stacked Autoencoder to learn the high-level features separately from the multimodal ROI features as those in (Zhang et al., 2011) and a multi-kernel SVM was used to combine these features to improve the classification performance. A deep learning framework based on 3D convolutional neural networks (CNN) was proposed for estimating missing PET imaging data with respect to the MRI data, and then the voxel-wise GM density map of MRI and the intensity values of PET are combined with a sparse regression classifier for multimodal classification of AD (Li et al., 2014).

Although the above methods have shown their effectiveness in AD diagnosis, there are still some problems to solve in these methods. The ROI-based feature extraction may ignore some minute abnormal changes although it can significantly reduce the feature dimension and provide robust representations. In addition, the ROIs are generated by the prior hypotheses and the abnormal brain regions relevant to AD might not fit well to the pre-defined ROIs, thus limiting the representation power of extracted features. Since the ROI method need to extract the influential brain regions, complex registration and segmentation are often required in image preprocessing. The voxel-wise features can alleviate this problem, but they are of huge dimensionality, far more features than training subjects, which may lead to low classification performance due to the ‘curse of dimensionality’. In addition, the extraction of these handcrafted features requires domain expert knowledge. The success of the above CAD methods highly depends on the performance of the image preprocessing, including rigid registration and segmentation. Thus, it is still a challenging task to automatically learn the latent and generic features and perform the classification for AD diagnosis using PET images.

Recently, deep learning methods such as CNN have been widely investigated for image classification and computer vision (Simonyan and Zisserman, 2014; Ng et al., 2015; Chen et al., 2016; Wang et al., 2017). Deep CNN has been successfully applied to jointly learn the image features and discrimination for object detection and image analysis with less image preprocessing (Simonyan and Zisserman, 2014). The deep 3D CNNs have been studied to extract the features of 3D medical images for classifications (Hosseini-Asl et al., 2016). In addition, recurrent neural network (RNN) was investigated for learning the features of sequential images such as video classification and video action recognition (Cabral et al., 2013; Zhan et al., 2015). The deep 2D CNNs and Long Short-Term Memory (LSTM) networks were combined to capture the spatial and time sequential features of video for action recognition (Zhan et al., 2015). In addition, RNN has been investigated to model the 3D structure of medical images for image segmentation (Chen et al., 2016). The intra-slice and inter-slice context information are exploited by a combination of a fully convolutional network (FCN) and a RNN for 3D biomedical images segmentation.

Since 3D CNNs can capture the 3D structure of brain image better than 2D CNNs, one direct way for image classification is to construct a 3D CNN for extraction of 3D spatial features from the 3D PET images. But the 3D PET brain images are of large size (256 × 256 × 256 voxels in our experiments), which requires to train a deeper CNN to capture the rich structure information. Different from the computer vision, the brain image set for AD diagnosis is usually small. Overfitting is still a critical challenge in training a deeper CNN model with a limited number of training samples compared to the large number of learnable parameters (Shen et al., 2017). This will result in low performance of deep models. Instead, this paper proposes a new classification framework based on combination of 2D CNN and RNN, which learns the features of 3D PET images for AD diagnosis. In this framework, the 3D image is decomposed into a sequence of 2D image slices based on a single view. Then, the deep 2D CNNs are built to capture the intra-slice features while RNN is used to extract the inter-slice features. The BGRU is used in modeling RNN. Finally, both the intra-slice and inter-slice features are integrated for final classification of 3D PET images. The proposed method investigates the combination of CNNs and BGRU to learn more generic features for classification. To our best knowledge, there are few studies focusing on combination of CNN and RNN for 3D medical image classification. Compared to the 3D CNN, the proposed method has the following advantages. First, the 3D PET image is decomposed into a sequence of 2D images to train a 2D CNN, which can reduce number of learnable parameters and increase the number of training samples. Thus, the 2D CNN model is easier to train for extraction of the intra-slice features. Second, RNN is further applied to extract the inter-slice features, and both the intra-slice and inter-slice features are jointly learned and integrated to capture the rich 3D spatial features for improving the image classification.

The remainder of this paper is organized as follows. In next section, we will present the image set used in this work and the proposed method. Then we will present the details of experimental results and discussion. Finally, we will conclude this paper.

## Data Set and Method

**FIGURE 1:**
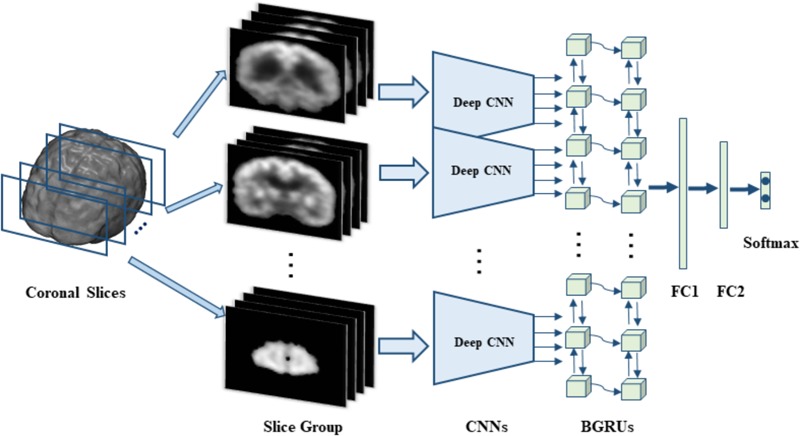
The flowchart of our classification framework for the sagittal direction, we first decompose 3D PET image into many groups of slices and then build a deep CNN to capture the intra-slice features for each group while two stacked BGRU layers are trained to capture the inter-slice features. On top of BGRUs, two fully connected layers (FC1 and FC2) and one softmax layer are added to make classification prediction.

In this section, we will present the data set used in this work and the proposed method in detail. Our proposed method makes no assumption on a specific neuroimaging modality. The 3D FDG-PET image is a powerful imaging modality for AD diagnosis and is studied in this work. We propose a deep learning framework based on combination of CNN and BGRU for AD diagnosis using 3D FDG-PET images. The complex problem of 3D image classification is decomposed into the ensemble classification of 2D slice images. **Figure 1** shows the flowchart of our proposed framework for a single direction of 3D PET images. The CNN and BGRU are cascaded and combined to learn the intra-slice and inter-slice features of 3D PET images for classification prediction. The proposed method consists of three main steps: image preprocessing and decomposition, intra-slice feature learning by deep CNN, inter-slice feature learning by BGRU, and final ensemble classification, as detailed in the following subsections.

### Data Set

In this study, all data set were obtained from the Alzheimer’s Disease Neuroimaging Initiative (ADNI) database, which are publicly available in the website^1^. The ADNI was launched in 2003 by the National Institute on Aging (NIA), the National Institute of Biomedical Imaging and Bioengineering (NIBIB), the Food and Drug Administration (FDA), private pharmaceutical companies and non-profit organizations, as a $60 million, 5-year public–private partnership. The primary goal of the ADNI was to test whether serial magnetic resonance imaging (MRI), PET, other biological markers, and clinical and neuropsychological assessments can be combined to measure the progression of MCI and early AD. Determination of sensitive and specific markers of very early AD progression is intended to aid researchers and clinicians to develop new treatments and monitor their effectiveness, as well as reduce the time and cost of clinical trials. The principal investigator of this initiative is Michael W. Weiner, M.D., VA Medical Center and University of California, San Francisco. ADNI was the result of efforts of many co-investigators from a broad range of academic institutions and private corporations. The study subjects were recruited from over 50 sites across the United States and Canada and gave written informed consent at the time of enrollment for imaging and genetic sample collection and completed questionnaires approved by each participating sites Institutional Review Board (IRB).

#### Subjects

In this work, we use the 18-Fluoro-DeoxyGlucose PET (FDG-PET) imaging data from the baseline visit of ADNI database for evaluation. These imaging data was acquired from 339 ADNI participants including 93 AD subjects, 146 MCI subjects (76 MCI converters (pMCI) and 70 MCI non-converters (sMCI)), 100 NC subjects. **Table 1** presents the demographic details of the studied subjects in this paper.

**Table 1:** Demographic characteristics of the studied subjects (from ADNI database).

Diagnosis	Number	Age	Gender (F/M)	MMSE	Education	CDR
AD	93	75.49 ± 7.4	36/57	23.45 ± 2.1	14.66 ± 3.2	0.8 ± 0.25
MCI	146	75.35 ± 6.7	49/97	26.99 ± 1.8	15.48 ± 2.7	0.5 ± 0.04
NC	100	75.93 ± 4.8	39/61	28.93 ± 1.1	15.83 ± 3.2	0 ± 0

*CDR: the Clinical Dementia Rating. MMSE: the Mini-Mental State Examination. The values are denoted as mean ± standard deviation.*

#### FDG-PET Data Acquisition and Preprocessing

In ADNI, PET image acquisition had been done according to the ADNI acquisition protocol in (Jack et al., 2008). The FDG-PET images were acquired by 30–60 min post-injection, and were averaged, spatially aligned, interpolated to a standard voxel size, normalized in intensity, and smoothed to a common resolution of 8 mm full width at half maximum. More detailed information about the FDG-PET acquisition procedures is available at the ADNI website. Each image was examined for major artifacts, and its orientation was adjusted if necessary.

The FDG-PET images were processed to make the images from different systems more similar. The processing included affine registration to a template, intensity normalization, and conversion to a uniform isotropic resolution of 8 mm FWHM as those in (Silveira and Marques, 2015). No image segmentation and rigid registration are required for the images. For consistency, all images were resampled to size of 256 × 256 × 256 and resolution of 1 × 1 × 1 mm^3^. The voxels outside the brain are removed from the image analysis, and the FDG-PET images are reduced to size of 193 × 153 × 163 for classification.

In this work, the complex classification problem of 3D images is divided into a number of 2D image classification problems and their integrations. Thus, each 3D FDG-PET image is decomposed into a number of 2D slices along the third direction (e.g., axials, sagittal, and coronal). Since there is no rigid registration performed on the image, the adjacent slices may have similar structure. The decomposed slice images are partitioned into a few number of groups at a certain interval with some overlaps. Each group consists of 15 slices and interval is set to 9 slices in our experiments. For each group of slices, a deep 2D CNN network is built and trained to capture the intra-slice features. Since all deep 2D CNN network shares the same structure, all the decomposed 2D slices is reduced to the same size of 150 × 150 by cutting off the peripheral to facilitate building CNN. To extract the inter-slice features, two stacked BGRUs are constructed with a sequence of input feature vectors, which are generated from the sequential 2D image slices. The features generated by BGRU are further fed into two stacked full connection layers followed by a softmax layer to make the classification prediction.

### Learning Intra-Slice Features by Deep CNN

Different from the conventional methods that explicitly extract the regional and handcrafted features, deep CNN is used to learn the latent and generic features from the 2D image slices. CNN is a special kind of multi-layer neural networks, which has been widely used in image classification and object detection (Lécun et al., 1998; Krizhevsky et al., 2012; He et al., 2015). It is designed to recognize visual patterns with extreme variability directly from pixel images with minimal preprocessing and with the robustness to distortions and simple geometric transformations. To extract the intra-slice features, we propose to build the 2D CNN models for learning intra-slice characteristics invariant to the simple distortions and geometric transformations. For each group of slices, the 2D CNN model is constructed and trained with the slices from the same group. There are several variations on the CNN architecture. Typically, a deep CNN for feature extraction alternatively stacks several convolutional and sub-sampling layers followed by fully connected layers. In this work, the 2D CNN architecture is composed of convolutional, max pooling, full connection and softmax classification layers as shown in **Table 2**. **Table 2** also lists the details of the relevant parameters for the architecture of deep 2D CNNs. For simplification, the same network structure is used to build the deep CNNs for all groups of slices, but with different parameters trained for different groups.

**Table 2:** The network architecture and parameters of our deep CNNs.

Layer ID	Layer name	Kernel number	Kernel size/Stride	Output size
0	Input			1 × 150 × 150
1	Conv1	16	3 × 3/2	16 × 74 × 74
2	Conv2	16	3 × 3/1	16 × 72 × 72
3	Max-Pooling 1		2 × 2/2	16 × 36 × 36
4	Conv3	32	3 × 3/1	32 × 34 × 34
5	Conv4	32	3 × 3/1	32 × 32 × 32
6	Max-Pooling 2		2 × 2/2	32 × 16 × 16
7	Conv5	64	3 × 3/1	64 × 14 × 14
8	Fatten			12544
9	Full-Connected 1	512		512
10	Full-Connected 2	256		256
11	Softmax	2		2

The first type of layer is the convolutional layers which convolve the learned filters with the input image, followed by adding a bias term and applying a non-linear activation function, and finally generate a feature map for each filter. Through convolution, the features can capture the discriminatory information of the image. The second layer is the max-pooling layers which down-sample the input feature map along the spatial dimensions by replacing each non-overlapping block with its maximum. Max-pooling can help to keep the most influential features for distinguishing images. Through max-pooling, the features become more compact and efficient from low to higher layers, which can achieve robustness to some distortions and geometric variations such as shift, scale and rotation at a certain level. The third type of layer is the fully connected layers, which takes all neurons in the previous layer and connects them to every output neuron. Each output neuron produces the learned linear combination of all inputs from the previous layer and passed through non-linearity. Finally, a softmax classification layer is appended to the last fully connected layer and is fine-tuned back-propagation with negative log-likelihood to predict class probability. The softmax function is a derivation of logistic function that highlights the largest values in a vector while suppressing those that are significantly below the maximum. The outputs of the softmax layer can be interpreted as class prediction probabilities from 0 and 1. The sum of all the outputs is always 1.

In our implementation, each deep CNN is composed of 5 convolutional layers, 2 max pooling layers, 2 fully connected layers and 1 softmax layer, as shown in **Table 2**. The sizes of all convolutional filters are set to 3 × 3 and the filter numbers are set to 16, 16, 32, 32, and 64 for 5 convolution layers, respectively. Max-pooling is applied on each 2 × 2 region. *Tanh* function is adopted as the activation function in these layers. The convolutional kernels are randomly initialized on the Gaussian distribution. The other trainable parameters of the networks are tuned using the standard back-propagation with stochastic gradient descent (SGD) by minimizing the cross-entropy loss. The dropout strategy for regularization (Srivastava et al., 2014) is employed to reduce the co-adaption of intermediate features and overfitting problem, and then improve generalization capability. In the training stage, each deep CNN is trained separately to capture the specific features in the group of image slices for the classification task. Therefore, each image slice is converted into a high-level feature vector from the last fully connected layer for the following processing.

### Staked BGRUs for Learning Inter-Slice Features

**FIGURE 2:**
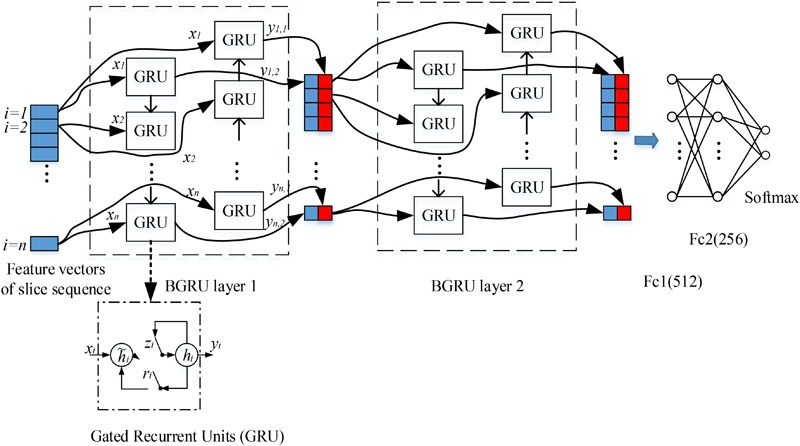
Our deep recurrent neural network (RNN) based on two stacked layers of bidirectional gated recurrent units (BGRU), where *xi* are the feature vectors generated from image slice *i* as the inputs of BGRU while *yi1 and yi2* are the output feature vectors of GRU from two directions (the internal structure of GRU is shown in the bottom, where *ri, zi, hi* and *hi* are the reset gate, update gate, candidate hidden layer and output layer, respectively). On top of stacked BGRU layers, two fully connected layers and one softmax layer are appended for classification prediction.

The 3D FDG-PET image is considered as a set of 2D slice sequences in this work. Recurrent neural networks (RNNs) are effective to process and model the sequential data. While the intra-slice features are extracted with the deep 2D CNNs, RNN is applied to capture the inter-slice features of the 3D images. We propose the stacked RNNs to model the sequential correlations between the consecutive slices and extract the inter-slice features as shown in **Figure 2**.

In the past decades, the performance of RNN was severely restricted due to the difficulty of training. Gradient mass and explosion are the common unsolved problems until the emergence of a special kind of RNN structure—LSTM. LSTM contains three gate units (*forget* gate, *input* gate and *output* gate) and a memory cell unit. By updating the state of memory cell through three gates, LSTM can discard irrelevant information and effectively capture the helpful information in sequence. Gate recurrent unit (GRU) was proposed as a special kind of variants for LSTM (Cho et al., 2014). Through removing the memory cell from the original LSTM, GRU makes RNN simpler without degrading performance. In (Chung et al., 2014), the author made a detailed comparison of these two kinds of RNN architectures and concluded that GRU has a slightly better performance than LSTM.

Compared to LSTM, GRU has only two gates: *update* gate *z* and *reset* gate *r*, thus it has the advantages of less parameters and easier training. The *forget* and *output* gates of LSTM are merged into a single *update* gate *z*, which is used to get the current state of the output via linear interpolation in GRU. When the input is (*x*_i_, *h*_i-1_), which denote the input features of the *i*^th^ image slice and the previous hidden state, the *update* gate *z* and *reset* gate *r* are computed as:

(1) $$z_{i}=\sigma (W^{xz}x_{i}+W^{hz}h_{i-1})$$

(2) $$r_{i}=\sigma (W^{xr}x_{i}+W^{hr}h_{i-1})$$

where *W*^xz^, *W*^hz^, *W*^xr^ and *W*^hr^ are the corresponding weight matrices; σ is a logistic sigmoid function. The candidate state of the hidden unit is computed by:

(3) $$\;{\tilde{h}}_{i}=\tan(W^{xh}x_{i}+W^{hh}(h_{i-1}⊙r_{i}))$$

where ⊙ is an element wise multiplication. When *r*_i_ is close to 0 (off), the reset gate *r* effectively makes the unit to read the first symbol of an input sequence and forget the previously computed state. The *i^th^* hidden activation state *h*_i_ of GRU is a linear interpolation between the previous state *h*_i-1_ and the candidate state 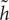
_i_:

(4) $$h_{\text{i}}=(1-{\text{z}}_{\text{i}})⊙{\tilde{h}}_{\text{i}}+{\text{z}}_{\text{i}}⊙h_{\text{i}-1}$$

Each image slice has no specific direction and is correlated with both its preceding and following slices in capturing the discriminative information. Thus, we apply the Bidirectional-GRU (BGRU), which consists of a forward GRU (*i* is from 1 to *n*) and a backward GRU (*i* is from *n* to 1), to capture more correlation features between the image slices. The inputs of each BGRU include the features of image slices produced by 2D CNNs. For each group, feature vectors are generated from 2D image slices extracted at a certain interval from the 3D FDG-PET image. To capture more detailed inter-slice information by BGRU without sacrificing the efficiency, each group contains 15 image slices decomposed from a 3D image for training one deep 2D CNN. The image groups are partitioned at an interval of 9 slices and with an overlap of 6 slices between the neighboring groups. The outputs of both forward and backward GRUs are concatenated together to form the outputs of BGRU at the same time. In addition, to further enhance the inter-slice information flow in the network, two layers of BGRUs are stacked into a deep structure by taking the outputs of one BGRU as the inputs of another BGRU. **Figure 2** shows the inter-slice learning model by stacked BGRUs for all groups of image slices. To alleviate overfitting problem, the dropout techniques are also adopted in training the BGRU network of RNN (Gal and Ghahramani, 2016). At last, the learned features from the outputs of BGRUs are concatenated together as the inputs to the fully connected and softmax layers for the classification task.

### Final Ensemble Classification

For image classification task, the BGRU network layer is followed by two fully connected layers and one softmax layer. The features generated from the second BGRU layer are fed into the followed two fully connected layers, and a softmax layer is appended for final classification prediction. The BGRU, the fully connected, and the softmax layers are jointly optimized for classification. Finally, we can obtain the classification prediction probability for each subject. Training of the proposed CNN-GRU classification framework consists of pre-training of individual 2D CNNs, and fine-training of the stacked GRU networks for the task-specific classification. Initially, each deep 2D CNN is individually pre-trained for each group of 2D image slices by directly mapping the outputs of fully connected layer to the probabilistic scores of class labels with softmax function. Then, the initial-trained parameters of 2D CNNs are fixed for all convolution and pooling, and fully connected layers, while the parameters of the GRUs are trained jointly with the upper 2 fully connected and softmax prediction layers. The training iteration ends when the validation error rate stops decreasing.

**FIGURE 3:**
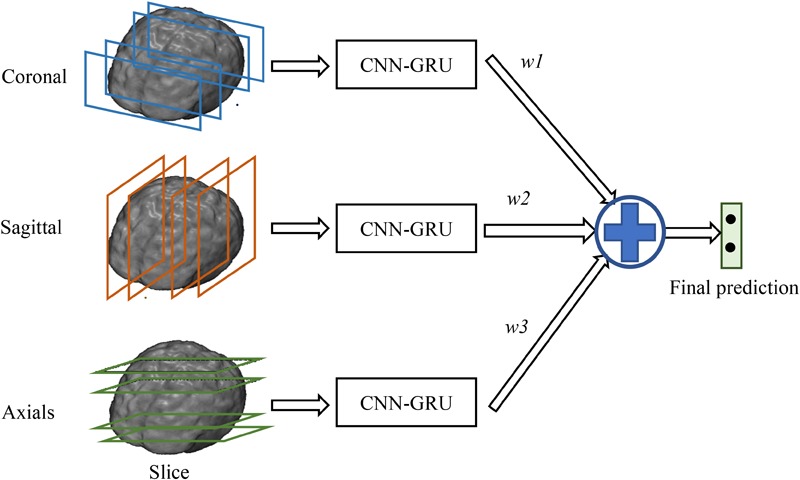
Final ensemble of multi-view classifications.

In addition, since the decompositions of 3D FDG-PET images at the axial, sagittal and coronal directions may capture the complementary information for classification from different views of the 3D context. Thus, we train individual CNNs and BGRU combination models (CNN-GRU) for axial, sagittal and coronal directions and obtain three prediction scores. The final classification is performed by weighted averaging three prediction scores obtained from three different views as shown in **Figure 3**. The weights are determined by grid search of the possible values for fusion of three direction BGRU prediction scores on the validation set.

### Feature Sets and Implementation

To learn the features with 2D CNN, the 3D FDG-PET image is first decomposed into a number of 2D image slices. In total, there are 193 image slices decomposed in coronal direction, 153 slices in sagittal direction and 163 slices in axial direction for each subject. The decomposed image slices are further partitioned into a number of groups at an interval of 9 slices and with an overlap of 6 slices. Thus, each group for a subject consists of 15 image slices for training one deep 2D CNN. Therefore, there are 20 deep CNNs in coronal, 17 CNNs in sagittal and 18 CNNs in axials obtained to generate the intra-slice feature vectors for 3D FDG-PET images. After training of 2D CNNs, the CNNs with low classification accuracies on the validation sets are removed and not considered for further processing. For each group of slices, the features generated from 4 image slices are fed into the inputs of BGRU. The number of hidden units in each BGRU is set to 256.

Our proposed algorithm is implemented with the Keras library (Chollet, 2015) in Python, which is based on Theano (Bastien et al., 2012). The experiments are conducted on PC with a NVIDIA GeForce GTX TITAN X GPU with 12GB memory. The initial weights of the deep network are set as the defaults in Keras, i.e., Xavier uniform. For training the deep CNNs, SGD is used with a low learning rate of 1 × 10^-4^, and momentum is set to 0.9 and weight decay is set to 1 × 10^-6^. The deep networks are stable after iteration of about 100 epochs. Adam optimizer algorithm (Dauphin et al., 2015) is adopted for training the BGRU. The batch sizes for training CNN and GRU are set to 40. Validation set is used for optimization of the training process. To avoid overfitting problem, dropout technique, L1 and L2 regulations are used in our deep networks (Srivastava et al., 2014). *Relu* activation is applied for each neuron of 2D CNN while *tanh* activation is used for the BGRU hidden unit.

In our experiments, we will evaluate the effectiveness of deep CNNs and BGRU on the classification. First, we test the classification performance of each individual CNNs and select the best CNNs with the highest classification accuracy from three different directions. Second, we evaluate the classification performances after adding the BGRU to combine the intra-slice features from each direction. Finally, we present the classification performance of the proposed method by weighted averaging of prediction scores at three directions.

## Experimental Results

The proposed algorithm was tested on the classifications of AD vs. NC and MCI vs. NC. The datasets that we used in the experiments are from the ADNI dataset and the image preprocessing is conducted as illustrated in the above section. The FDG-PET brain images are captured from 339 participants including 93 AD subjects, 146 MCI subjects and 100 NC subjects. Ten-fold cross-validation is performed to reduce the influence of random factors. Each time, one fold of the image set is used for testing; another fold is used for validation while the left eight folds are used for training. The validation set is used for monitoring the training process to obtain the model weights with the optimized performance. The classification performances are averaged on the test set of 10 folds. To evaluate the classification performance, we compute four measures for comparison in the experiments, which are classification accuracy (ACC), sensitivity (SEN), specificity (SPE), receiver operating characteristic curve (ROC) and area under ROC (AUC). The ACC is defined as the proportion of correctly classified samples among the total number of samples. SEN is computed as the proportion of correctly classified positive samples (patients) among the total number of positive samples. The SPE is computed as the proportion of correctly classified negative samples (Normal Controls) among the total number of negative samples. The ROC curves plotting the true positive rate (TPR) against the false positive rate (FPR) at various threshold settings are also shown and the AUC are computed for more comparisons.

### Test the Effectiveness of Deep CNNs and BGRU

**Table 3** shows the performance comparisons of the deep CNNs and BGRU on classifications of AD vs. NC and MCI vs. NC. The ACC and AUC are used to evaluate the classification performance. The best 2D CNNs with the highest classification accuracy from three different directions are denoted as “Coronal-CNN,” “Sagittal-CNN” and “Axial-CNN,” respectively. Adding BGRU to combine the intra-slice features from three directions are denoted as “Coronal-BGRU,” “Sagittal-BGRU” and “Axial-BGRU,” respectively. Our proposed method with weighted averaging of prediction scores at three directions is denoted as “Weighted Fusion” in **Table 3**.

**Table 3:** The performance comparison of deep CNNs, BGRU and their combination.

Task	AD vs. NC (%)		MCI vs. NC (%)	
	AUC	ACC	AUC	ACC
Coronal-CNN	91.8	84.5	79.8	71.2
Sagittal-CNN	91.2	85.0	77.9	74.4
Axial-CNN	91.4	84.5	78.7	74.0
Coronal-BGRU	94.6	88.6	80.8	74.8
Sagittal-BGRU	94.8	90.7	82.5	77.6
Axial-BGRU	94.7	88.1	83.1	76.0
Weighted Fusion	95.3	91.2	83.9	78.9

**FIGURE 4:**
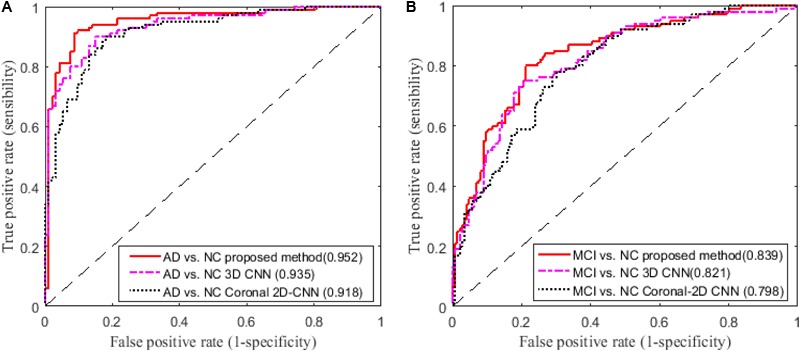
Comparisons of ROC curves on classification of **(A)** AD vs. NC and **(B)** MCI vs. NC (AUC is given in brackets).

In addition, we compare our proposed method to the deep 2D CNN and 3D CNN methods. The results of 2D CNNs are based on the Coronal-CNN which performs best among all 2D CNNs. We build a deep 3D CNN on the same data set to learn the latent and generic features for image classification. The architecture and parameters of the deep CNN are optimized with our best efforts. **Table 4** shows the performance comparisons of the 2D CNN and 3D CNN as well as our proposed method on classifications of AD vs. NC and MCI vs. NC. **Figures 4A,B** display the comparisons of their ROCs for classifications of AD vs. NC and MCI vs. NC, respectively. The ACC, SEN, SPE, ROC and AUC are used to evaluate the classification performance.

**Table 4:** Comparison of the classification performances with 2D/3D CNNs and the proposed method.

Method	AD vs. NC (%)				MCI vs. NC (%)			
	ACC	SEN	SPE	AUC	ACC	SEN	SPE	AUC
2D-CNN	84.5	82.8	86.0	91.8	71.2	79.5	51.0	79.8
3D-CNN	87.1	87.1	84.0	93.5	75.6	82.2	66.0	82.1
Our method	91.2	91.4	91.0	95.3	78.9	78.1	80.0	83.9

*SEN = TP/(TP + FN); SPE = TN/(TN + FP). TP: true positive; TN: true negative; FP: false positive; FN: false negative.*

### Comparison With Existing Methods

In this section, we compare our results with some recent results reported in the literature which are also based on FDG-PET data from ADNI database for AD and MCI diagnosis. In particular, the results reported in three recent methods (Gray et al., 2011; Li et al., 2014; Silveira and Marques, 2015) are compared with our results in **Table 5**, as briefly described following. In (Silveira and Marques, 2015), a boosting classification method, which effectively combined several simple classifiers and performs feature selection concurrently with the segmentation, was proposed to improve the classification performance when compared with the SVM classifier. A multi-region analysis based on anatomical segmentations, which are automatically generated in the native space of each subject instead of the space of a single reference image, was proposed for extraction of regional signal intensities and a SVM classifier was used for feature classification in (Gray et al., 2011). In (Li et al., 2014), the voxel-wise intensities of FDG-PET neuroimages were extracted as features after performing the rigid registration to the respective MR images and a sparse regression classifier was used for feature classification. The above methods include the traditional extractions of ROI and voxel-wise features and the ensemble classification on FDG-PET images from ADNI database. But their codes are not released and it is not easy to implement these published methods on the same settings for fair comparison. Thus, we use the reported results in the literature for comparison of these methods in **Table 5**.

**Table 5:** Comparison of the classification performances reported in the literature.

Method	AD vs. NC (%)				MCI vs. NC (%)			
	ACC	SEN	SPE	AUC	ACC	SEN	SPE	AUC
(Silveira and Marques, 2015)	90.9	–	–	–	79.6	–	–	–
(Gray et al., 2011)	81.6	82.7	80.4	90.0	70.2	73.8	62.3	73.0
(Li et al., 2014)	–	–	–	89.8	–	–	–	70.1
Our method	91.2	91.4	91.0	95.3	78.9	78.1	80.0	83.9

*SEN = TP/(TP + FN); SPE = TN/ (TN + FP). TP: true positive; TN: true negative; FP: false positive; FN: false negative.*

## Discussion

From the results in **Table 3**, we can observe that BGRU can boost the classification performance in all three directions for both classification tasks of AD vs. NC and MCI vs. NC. This demonstrates that the inter-slice sequential features can provide complementary information and improves the classification performance for FDG-PET images. We also found that the sequence of image slices in the sagittal direction have the best ACC and AUC by using the BGRU for AD classification. Nevertheless, the fusion of prediction scores from three directions can further enhance classification performance. From the results of **Table 4** and **Figure 4**, we can see that the 3D CNN performs better than the 2D CNN. This is because the 3D CNN can capture more information about the 3D spatial context for classification. These results also demonstrate our proposed algorithm can achieve better performance than the 3D CNNs on classification of AD and MCI subjects from NC subjects. The proposed CNN-BGRU combination framework can more effectively learn the discriminative features than 3D CNNs.

From the results in **Table 5**, we can see that our proposed method performs better than other published methods. It is worth noting that the result differences may be caused not only by using different feature extraction and classification methods, but also with different ADNI subjects. In addition, the differences in the test samples, the partition of cross-validation to separate the training and testing sets can also make the fair comparison difficult to achieve. Different from the conventional methods that are based on the handcrafted ROI features (Gray et al., 2011; Silveira and Marques, 2015), the proposed method combines the 2D CNNs and RNN to automatically and hierarchically learn the features of 3D PET images for AD classification. Compared to the conventional methods, the proposed method has the following three main advantages. First, the feature extraction is a machine learning based method by learning deep CNNs, which is data-driven method and does not require any domain expert knowledge. Second, the intra-slice and inter-slice features are jointly learned and integrated to capture the rich 3D spatial features and improve the image classification. Third, no rigid registration or segmentation procedures are required in image processing, which can simplify the diagnosis procedure and save the computation costs. But there are still several weaknesses in the proposed method. First, it is not easy to optimize the parameters of the deep CNN, such as the number of layers, the size and number of kernels in each layer. Second, the learned features by the proposed method have no sufficient clinical information for visualization and interpretation of the brain neurodegenerative disease (i.e., AD or MCI) in the clinical application.

**FIGURE 5:**
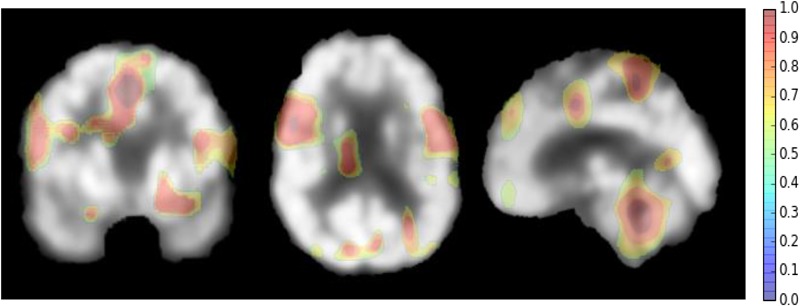
The network attention areas for AD diagnosis.

However, there are some suggestions to address the above weaknesses. For setting the parameters of the deep CNN, large kernel size is effective to capture the large patterns of PET image but it may ignore the small ones. More network layers can be used to learn the hierarchical and rich features. Thus, the size of all kernels is set to 3 × 3, but multiple convolutional layers are used to hierarchically capture the large patterns. The other optimal parameters can be obtaine by cross validation in our experiments. Since AD can affect the certain pathological patterns, only a subset of image regions may be closely related to AD diagnosis. It is also important to detect these regions for diagnosis and interpretations of the diseases. To achieve this, we attempt to systematically occlude different local regions of the input 2D image slice with a black square (25 × 25 voxels in our experiments), and monitoring the prediction outputs of classifier as a function of occluded positions as in (Zeiler and Fergus, 2014). When the occluded part covers the important regions related to AD, the probability of the correct class in the classifier output will drop significantly. In this way, we can generate a network activity map by evaluation of classification performance reduction as a function of occluded position. In our experiments, the top 3 groups of 2D image slices with the better classification performance are selected from the low-level 2D CNNs of three different directions. Then, the network attention areas are generated by compiling the prediction masks obtained by obstructing these selected image patches of 25 × 25 and measuring the drop of the output probability. This heatmap shows how the network learns the importance of local areas in prediction of disease status. The areas with the highest attention for FDG-PET images in AD diagnosis are demonstrated in **Figure 5**. These areas seem to be consistent with the ones that are mostly affected by AD, mainly in hippocampus, posterior temporal lobe and parietal lobe, the posterior cingulate gyrus and left parahippocampal gyrus (Zhang et al., 2011; Liu et al., 2015).

## Conclusion

In this paper, we have proposed a new classification framework based on combination of CNN and BGRU to capture the rich features of 3D FDG-PET images for AD diagnosis. The 3D FDG-PET image is decomposed into a set of 2D image slices and multiple deep 2D CNNs are built to learn the intra-slice context features while BGRU is applied to capture the inter-slice features of the 3D image context. No segmentation and rigid registration are required for preprocessing the FDG-PET image in the proposed method. The experimental results on the ADNI dataset have shown that the proposed method has achieved promising performance for classifications of AD and MCI.

## Author Contributions

ML made the main contribution of the paper including generating the main idea, conducting the experiments and writing the papers. DC made many contributions, including the implementation of the idea and revising the paper. WY contributed to the discussion of the methods and revising the papers.

## Conflict of Interest Statement

The authors declare that the research was conducted in the absence of any commercial or financial relationships that could be construed as a potential conflict of interest.

## Footnotes

1 http://adni.loni.usc.edu/
